# The affordable care act and family planning services: the effect of optional medicaid expansion on safety net programs

**DOI:** 10.1186/s40834-016-0029-y

**Published:** 2016-10-03

**Authors:** Bethany G. Lanese, Willie H. Oglesby

**Affiliations:** College of Public Health, Kent State University, 800 Hilltop Drive, 212 Moulton Hall, P.O. Box 5190, Kent, OH 44242 USA

**Keywords:** ACA, Health disparities, Public health, Inequality, Health policy

## Abstract

**Background:**

Title X of the Public Health Service Act provides funding for a range of reproductive health services, with a priority given to low-income persons. Now that many of these services are provided to larger numbers of people with low-income since the passage of the Affordable Care Act and Medicaid expansion, questions remain on the continued need for the Title X program. The current project highlights the importance of these safety net programs.

**Methods:**

To help inform this policy issue, research was conducted to examine the revenue and service changes for Title X per state and compare those findings to the states’ Medicaid expansion and demographics. The dataset include publicly available data from 2013 and 2014 Family Planning Annual Reports (FPAR). Paired samples differences of means t-tests were then used to compare the means of family planning participation rates for 2013 and 2014 across the different categories for Medicaid expansion states and non-expansion states.

**Results:**

The ACA has had an impact on Title X services, but the link is not as direct as previously thought. The findings indicate that all states’ Title X funded clinics lost revenue; however, expansion states fared better than non-expansion states.

**Discussion:**

While the general statements from the FPAR National surveys certainly are supported in that Title X providers have decreased in number and scope of services, which has led to the decrease in total clients, these variations are not evenly applied across the states. The ACA has very likely had an impact on Title X services, but the link is not as obvious as previously thought.

**Conclusion:**

Title X funded clinics have helped increase access to health insurance at a greater rate in expansion states than non-expansion states. There was much concern from advocates that with the projected increased revenue from Medicaid and private insurance, that Title X programs could be deemed unnecessary. However, this revenue increase has yet to actually pan out. Title X still helps fill a much needed service gap for a vulnerable population.

## Introduction

When it comes to improving the reproductive health of the population, the United States confronts multiple persistent issues. Almost half (49 %) of all pregnancies in the United States are unintended. This number is even higher for adolescent and young women, women of color, and women with low income and education levels [15]. With unintended pregnancies comes a greater risk of poor maternal and infant social, economic, and health outcomes [14].

Family planning is defined as “the ability to achieve desired birth spacing and family size.” Because of its impact on the health of infants, children, and women, family planning was rated as one of the top 10 overall achievements in public health in the 20th century [4]. The full scope of health care services that can be addressed during the delivery of family planning services can include medical services such as screening programs for cancer, STDs, educational programs on prevention, and various counseling programs [9].

The current project examines early changes in Title X funded family planning centers in the year following Medicaid expansion for 24 states under the Affordable Care Act. Title X service centers are often considered a safety net care provider for millions of low income people, with the vast majority being women. Since the passage of the ACA questions continue about the necessity of the services provided by Title X funding [3, 17]. The current project helps to illustrate the importance of these programs, and how they work in conjunction with Medicaid expansion to serve an additional need beyond health insurance. The Title X programs still provide safety net care for vulnerable populations.

## Background

Comprehensive family planning services provide a much needed benefit from an economic, health, and overall societal viewpoint. Preventive care with regard to reproductive health services has long-term and expansive effects [11]. Several preventive services are now offered under the Affordable Care Act with no cost sharing, and among these are contraceptives [18]. As stated in a brief published by the Center for American Progress, “Whether through reducing the cost of unintended pregnancies or enabling women to advance their education and careers, family planning provides women with greater independence to make crucial life decisions on their own terms—decisions that affect not only their lives but also the greater society,” [2].

Title X is a key source of public funding to support family planning services and Medicaid is an very important source of revenue at Title X service sites [1]. Under the Affordable Care Act, after the 2012 Supreme Court decision, states have the option to expand Medicaid coverage to non-parents under the age of 65 who have incomes below 138 % of the Federal Poverty Line [19]. By the end of 2014, 24 states and the District of Columbia had chosen to expand Medicaid [12]. The states that opted out of Medicaid expansion do not typically cover adults without children. Also, the income eligibility requirements are as low. For example, in Alabama it is 13 %, Texas is 15 %, Idaho is 24 %, and so on [13]. Alaska (129 %) is the only non-expansion state that has adult income criteria that is over 100 % of the Federal Poverty Line [13].

### Family planning services

A lingering challenge in the United States is improving the reproductive health of the U.S. population. Forty nine percent of all pregnancies are unintended, and the United States has one of the highest adolescent pregnancy rates of developed countries [9]. Preterm birth and infant mortality rates are also high in comparison to rates of other developed countries. Racial and ethnic minority populations are disproportionately impacted across all of these outcomes [5]. These challenges can be addressed by providing family planning services that include education, counseling, and medical services.

Family planning funding has a substantial impact on future expenditures for both the public and private sectors. It is estimated that there is a $4 savings in short-term public funding costs in medical care for every $1 spent to prevent unintended pregnancy [8]. Using 2010 Title X data, the Guttmacher Institute calculated that the average cost for a Medicaid-covered birth was $12,770, whereas one year of Title X clinic provided contraception was $269 per client [2]. The return is similar for private providers. However, in the United States, public support for providing the full range of reproductive health care services, including contraception, reproductive health related counseling, and cancer screenings remains a challenge.

### The Title X program

Title X of the Public Health Service Act provides funding for the provision of family planning services, which include various sexual and reproductive health services, including contraception [16]. It is the only federal grant program focused entirely on providing comprehensive family planning and related preventive health services, with priority given to low-income persons [16]. Questions have been presented about the necessity of Title X programs since the passage of the ACA, which has increased the number of insured persons and overall access to health services.

The Title X program is administered by the Office of Population Affairs (OPA) under the U.S. Department of Health and Human Services. They serve around 4 million clients annually through various service sites, including state, county, and local health departments. Community health centers, Planned Parenthood centers, other hospital, school, and faith- based programs, and private nonprofits also are among the Title X grantees (Office of Population Affairs 2015).

Research clearly shows health disparities related to sexual and reproductive health for low-income and women of color [5] and Title X programs have been successful at providing needed services to these populations. In addition, Title X clinics help Medicaid-eligible patients with enrollment and help other patients enroll in exchanges, which help to increase health care access. Title X staff are specifically trained in meeting the needs of vulnerable populations, like individuals with limited English proficiency, teenagers, and those confronting complex medical and personal issues such as substance abuse, disability, homelessness or intimate partner violence [16]. Title X funding goes toward supporting this infrastructure, such as personnel training, community education, and other such services [10].

According to the Title X Congressional Research Service report, the FY2016 Justification section of the Health Resources and Services Administration (HRSA), the Administration expected that Title X clinics would increase revenue because they would increase their proportion of clients with insurance coverage and the billing of third parties. However, the Family Planning Annual Report (FPAR) shows that overall dollar amounts of Title X revenue in 2014 actually declined. The size and reach of the provider service network has also had an overall decline. The overall decreases in Title X revenue from third parties (including Medicaid) and the size and reach of the provider network has again called into question the necessity and effectiveness of this program—especially after the implementation of the Affordable Care Act.

This research examines revenue and service changes in the Title X program on a state-by-state and regional basis and compares findings to state Medicaid expansion status and demographics to better explore the future role of Title X programs in a post-ACA health care marketplace. The research hypotheses are:

- Ho1: States that participated in Medicaid expansion will have a decrease in number of Title X clients served, encounters, and service sites, while states that did not participate will have an increase or no change.
- Ho2: States that participated in Medicaid expansion will have a decrease in the number of uninsured Title X clients served, while states that did not participate will have an increase or no change.
- Ho3: States that participated in Medicaid expansion will have an increase in revenue at Title X supported service sites, while states that did not participate will have a decrease in revenue or no change.

## Data and methodology

### Data

The dataset include data from 2013 and 2014 Family Planning Annual Reports (FPAR). All Title X family planning services grantees are mandated to submit FPAR data annually for monitoring and reporting program performance. The data are in summary form to maintain confidentiality of the clients who receive services at Title X funded service sites [6, 7]. The data are publically available, so ethical consent was not necessary.

The FPAR data include demographic information such as race, ethnicity, sex, age, and income level. It also provides information about the Title X grantees including the number of client encounters, the care providers, and the types of services obtained. Data are also collected regarding revenue source and client insurance.

### Methodology

Descriptive statistics were performed to describe the demographic details of the dataset by state. Paired samples differences of means t-tests were then used to compare the means of family planning participation rates for 2013 and 2014 across the different categories for Medicaid expansion states and non-expansion states. By the end of 2014, a total of 25 states plus the District of Columbia had participated in Medicaid expansion. Only those states that had already applied Medicaid expansion are included in the analysis. This includes the following 25 states, plus the District of Columbia: Arizona, Arkansas, California, Colorado, Connecticut, Delaware, District of Columbia, Hawaii, Illinois, Kentucky, Maryland, Massachusetts, Michigan, Minnesota, Nevada, New Hampshire, New Jersey, New Mexico, New York, North Dakota, Ohio, Oregon, Rhode Island, Vermont, Washington, and West Virginia.

The FPAR reports for 2013 and 2014 show the overall percentages and demographic information about Title X services users. For both years just over 90 % of all users were female, and around 70 % had family income levels at or below the federal poverty level--$23,850 for a family of four. Reports for both years show that the majority of Title X service users self-identified with at least one of the nonwhite Office of Management and Budget race categories (29 %) or as Hispanic or Latino (30 %). One major difference between the 2 years, however, is with the uninsured population. The 2013 report reveals that 63 % of users were uninsured, while in 2014 that number drops to 54 % [6, 7].

## Results

As shown in the FPAR National Summary, the overall number of clients receiving family planning services at Title X clinics decreased from 2013 to 2014 (See Table 1). There was an overall decrease of approximately 420,636 users. The 2014 National Summary shows that Title X number of clients, service sites, and project revenue have all decreased from 2013 to 2014. The revenue drop was approximately $71, 533 million net amount (constant 2014 dollars). While there was an increase in revenue from both private and other third-party payers, it was not enough to offset the losses from Medicaid of $27.6 million, a loss of almost $18 million in client services fees, a total loss of $28.8 million from state and local governments, $10.2 million from Title X, and a loss of $11.5 million from block grants and other revenue sources [7]. As shown in Table 1, there was a decrease in clients in all of the income brackets with the exception of the over 250 % of the federal poverty line (FPL).

**Table 1:** Title X family planning users 2013 and 2014

	2013	2014	Difference 2014-2013
Female Family Planning Users	4,146,861	3,734,418	(412,443)
Male Family Planning Users	370,724	362,531	(8,193)
US Total Family Planning Users	4,517,585	4,096,949	(420,636)
Total Number of Service Sites	4,168	4,127	(41)
Total Number of Encounters	8,170,151	7,215,032	(955,119)
Total Revenue (all sources)	$1,315,435,864	$1,243,901,947	($71,533,917)
Public health insurance	1,131,406	1,215,648	84,242
Private health insurance	453,535	559,845	106,310
Uninsured	2,865,672	2,239,377	(626,295)
Insurance status Unknown/not reported	107,211	114,413	7,202
Income Under 101 %	3,211,380	2,840,650	(370,730)
Income 101 to 150 %	636,484	572,948	(63,536)
Income 151 to 200 %	245,805	234,425	(11,380)
Income 201 to 250 %	103,246	100,402	(2,844)
Income Over 250 %	222,718	226,918	4,200
Income level Unknown/not reported	138,191	153,940	15,749

datasource: 2013 and 2014 FPAR National Summary

The FPAR regional reports list various reasons offered by the grantees as to why the demand for and use of Title X funded services went down, including clinical guideline changes, implementation of electronic health record systems and other impacts due to the ACA. We will discuss these factors in more detail in the discussion section.

It is expected that there will be differences in client numbers at Title X funded service centers due to the implementation of ACA provisions. For example, under the ACA, previously uninsured will have acquired health insurance, like those up to age 26 who are now allowed to stay on their parents’ plan. This will increase their options for healthcare providers; therefore, clients may then opt to get medical care from providers other than those funded through Title X grants.

The first research hypothesis states:

Ho1: States that participated in Medicaid expansion will have a decrease in number of Title X clients served, encounters, and service sites, while states that did not participate will have an increase or no change.

The findings, shown in Table 2, reveal that all states had a decrease in the number of Title X clients served. States that expanded Medicaid and those that did not both had a statistically significant decrease in total clients served and total number of client encounters. It is interesting that the number of service sites for states that did not expand Medicaid had a mean increase, while the states that did expand Medicaid showed an overall decrease in service sites.

**Table 2:** Medicaid expansion by state 2014 and 2013 differences clients, encounters, service sites

		Mean 2014	Mean 2013	Difference in Means	t-value
State did not expand medicaid	Total Title X Clients Served	62,345	70,253	−7908**	−2.84
	Total Title X Client Encounters	717,121	832,643	−115522***	−5.95
	Total Number of Title X Service Sites	566	540	26.3	1.30
State expanded medicaid	Total Title X Clients Served	96,321	104,871	−8550*	−2.29
	Total Title X Client Encounters	761,093	852,151	−91058***	−6.06
	Total Number of Title X Service Sites	383	394	−11.4	−1.22

datasource: 2013 and 2014 FPAR National Summary. *p < .05, **p < .01, ***p < .001

The FPAR Regional reports include more detailed discussion from the Title X grantees, including information as to what they think impacted the services in their area. Nearly all ten of the regional reports state the impact of ACA with regard to insurance as a factor, but there seems to be little consensus as to what impact it would have.

The second hypothesis is:

Ho2: States that participated in Medicaid expansion will have a decrease in the number of uninsured Title X clients served, while states that did not participate will have an increase or no change.

First, the results show an overall increase in the number of clients receiving services at Title X clinics being insured. From 2013 to 2014 there was a decrease of 626,295 uninsured clients for all service sites. In 2014 there were still approximately 54 % of all Title X clients who were uninsured; however, this number is down from 63 % who were uninsured in 2013. Table 3 shows that both states that did and did not expand Medicaid had a statistically significant decrease in the number of uninsured clients. There was a statistically significant increase in the number of clients with private insurance for states that expanded Medicaid. For states that did not expand Medicaid, the number of clients with private insurance also increased, but it was not statistically significant. Also, states that did not expand Medicaid showed a statistically significant decline in clients with public insurance, while states that expanded Medicaid showed an increase in the number of clients with public insurance. This difference was not statistically significant, but the directional difference is worth noting.

**Table 3:** Medicaid expansion by state 2014 and 2013 differences in insurance status

		Mean 2014	Mean 2013	Difference in Means	t-value
State Did Not Expand Medicaid	Client Insurance Status: Public Insurance	15,961	18,828	−2866*	−2.157
	Client Insurance Status: Private Insurance	10,371	8,520	1851	2.042
	Client Insurance Status: Uninsured	33,725	41,835	−8109*	−3.079
	Client Insurance Status: Unknown/Not Reported	2,288	1,071	1217	.981
STATE EXPANDED MEDICAID	Client Insurance Status: Public Insurance	30,315	24,562	5753	1.940
	Client Insurance Status: Private Insurance	11,268	8,986	2282**	3.243
	Client Insurance Status: Uninsured	52,591	68,397	−15806*	−2.521
	Client Insurance Status: Unknown/Not Reported	2,147	2,926	−779	−1.308

datasource: 2013 and 2014 FPAR National Summary. *p < .05, **p < .01, ***p < .001

As previously stated, the 2014 National Summary FPAR describes the overall decrease in revenue across all Title X funded service sites. There was a specific overall decrease in Medicaid revenue of $27.6 million. With Medicaid expansion, it is not clear if this reduction in Medicaid revenue was across all service sites, or if states that did not participate in expansion had a greater reduction. The third hypothesis is:

Ho3: States that participated in Medicaid expansion will have an increase in revenue at Title X supported service sites, while states that did not participate will have a decrease in revenue or no change.

The findings are displayed in Table 4, where it shows that states that did not expand Medicaid had a statistically significant (*p* < .01) decrease in Medicaid as a third party payer from 2013 to 2014. Also, states that did not expand Medicaid showed a statistically significant difference in overall revenue sources across all funding (*p* < .01). States that did expand Medicaid also showed a decrease, but neither categories were statistically significant. So while there was an overall decrease across all Title X funded sites, the decrease was greater for states that did not expand Medicaid.

**Table 4:** Medicaid expansion by state 2014 and 2013 differences: title x clinics revenue source medicaid revenue and total revenue

		Mean 2014	Mean 2013	Difference in Means	t-value
State did not expand medicaid	Clients with Medicaid as 3rd party payer	31,837,417	34,210,217	−2,372,800**	−3.307
	Total Overall Revenue all Sources	111,233,830	116,287,402	−5,053,571**	−3.195
State expanded medicaid	Clients with Medicaid as 3rd party payer	57,626,682	58,201,863	−575,180	-.702
	Total Overall Revenue all Sources	132,462,786	134,200,516	−1,737,730	−1.219

datasource: 2013 and 2014 FPAR National Summary. *p < .05, **p < .01, ***p < .001

## Discussion and limitations

While the general statements from the FPAR National surveys certainly are supported in that Title X providers have decreased in number and scope of services, which has led to the decrease in total clients, these variations are not evenly applied across the states. The ACA has very likely had an impact on Title X services, but the link is not as obvious as previously thought. The total number of clients decreased for states that did and did not expand Medicaid. This raises further questions as to where the women are going for these medical services in states that did not expand Medicaid, and how are they paying for them?

The FPAR data show that while there was a decrease in every Title X user income category under 250 % of the FPL, the over 250 % showed a slight increase. This supports the notion that there is a preference for Title X clinics by some users [17]. Some dependents who may not wish to bill their healthcare services to insurance because of confidentiality reasons or just simply due to care provider preferences will still seek care at Title X funded clinics.

The significant decrease in the number of uninsured clients in states that expanded Medicaid is very likely linked to the findings that the revenue decrease from 2013 to 2014 was not statistically significant. The increase in Title X clients with private health insurance in states that expanded Medicaid is certainly tied to the overt effort on the part of Title X clinics to assist with signing clients up for health insurance on the exchanges or with Medicaid. The Department of Health and Human Services (HHS) Office of Population Affairs awarded Title X enrollment assistance grants to 22 Title X service grantees in various states. Fifteen of the 22 grant recipient states are Medicaid expansion states. The purpose of the grants was to provide funding to initiate or expand outreach activities facilitating enrollment into health insurance. Title X service sites help eligible clients enroll into health insurance coverage through the Health Insurance Marketplaces, Medicaid, the Children’s Health Insurance Program (CHIP), or other local programs [16].

The FY2016 HRSA Justification stated that it was expected that Title X clinics would increase revenue in 2014 because of the increase in clients with health insurance and by billing third parties. This is shown to be partially correct. The Medicaid expansion states’ revenue did not increase, but the Non-Medicaid expansion states showed a statistically significant decrease in overall revenue. When compared to the outreach program grants and the overt attempts to sign up clients for health insurance, it seems the expansion states fared better.

The most obvious limitation of the present research is that it only contains one year of data post ACA implementation. When more data are available this research will be expanded. As of 2015, 29 states—an additional five from 2014—plus the District of Columbia have expanded Medicaid. Another limitation is that there are other factors outside of the ACA and not specific to Title X that have an impact on healthcare patterns. For example, the requirements for annual PAP tests have been changed to every 3 years, so this would impact the number of clients actually seeking services. As previously pointed out, the FPAR regional level reports offer detailed conjecture about the changes in client numbers from 2013 to 2014. All of the regional reports list factors related directly or indirectly to the ACA. Some of these include insurance changes, implementation of electronic health records, data collection issues, and initiatives under the ACA that clinics have to support outside of sexual and reproductive health, such as immunization programs. Other factors that the regional reports state involve staffing issues and changes, extreme weather conditions in 2014, and the increased preference of long-term birth control reflecting a national trend. Finally, there are certainly demographic differences across states that could impact upon the findings. Differences in average age among expansion states and non-expansion states could impact on the usage of a Title X clinics. There are also race and income differences between these states that could also play a role.

## Policy implications

Research not only shows that access to contraception dramatically reduces the likelihood of an unplanned pregnancy, but also that women with unintended pregnancies are less likely to have prenatal care. Those with unintended pregnancies are also more likely to engage in unhealthy activities, which lead to unhealthy babies with higher than average delivery and post-delivery care costs [8]. As stated in the FPAR reports, “Title X providers serve a vulnerable population, most of whom are female, poor, uninsured, and young,” ([7], executive summary).

As the current research shows, there are differences between states that expanded Medicaid and those that did not. These differences undoubtedly have an impact on access to care. The expansion states show a statistically significant increase in the number of clients with health insurance. Access to health insurance has clear implications on overall health outcomes and increasing access is one of the primary goals of the ACA. As shown here and in previous research Title X complements Medicaid [1, 10]. Not all low-income women are eligible for Medicaid, and Medicaid reimbursement may not cover the complete cost of services [10].

## Conclusion

Title X service sites provide sexual and reproductive health services with a focus on low-income women at reduced or no cost. There was great concern that with the projected increased revenue from Medicaid and private insurance, that Title X programs could be deemed unnecessary. However, this revenue increase has yet to actually pan out. Advocates pointed out that Title X supports many other things such as individual patient education, community-level outreach and public education about women’s health issues. Title X funding is also used to support infrastructure [1, 10]. These findings align with previous work in that “The continued provision of safety-net family planning services is important not just for the individual clients accessing services at these organizations but for broader health equity goals as well,” ([3], 60). Overall, it does seem that Title X funded clinics help fill a much needed gap in providing sexual and reproductive health services to vulnerable populations, even with the passage of the ACA.

### Overview

This research examines revenue and service changes in the Title X program compared to state Medicaid expansion status and demographics to determine the continued need for the Title X program.

## Abbreviations

ACA, Affordable Care Act; CMS, Centers for Medicare and Medicaid; FPAR, Family Planning Annual Reports

## References

[1] August E, Steinmetz E, Gavin L, Rivera MI, Pazol K, Moskosky S, Weik T, Ku L (2016). Projecting the unmet need and costs for contraception services after the affordable care act. Am J Public Health.

[2] Barry, D., Esenstad, A. (October, 2014). Ensuring access to family planning services for all. Center for American Progress. Available: https://cdn.americanprogress.org/wp-content/uploads/2014/10/FamilyPlanning-brief.pdf. Accessed 10 Oct 2015.

[3] Carter M, Desilets K, Gavin L, Moskosky S, Clark J (2014). Trends in uninsured clients visiting health centers funded by the title X family planning program — Massachusetts, 2005–2012. Morbid Mortal Wkly.

[4] CDC: National Center for Chronic Disease Prevention and Health Promotion (1999). Achievements in Public Health, 1900–1999: Family Planning. MMWR.

[5] Center for Reproductive Rights. (2010). Report on the United States’ Compliance with Its Human Rights Obligations in the Area of Women’s Reproductive and Sexual Health. Submission to the United Nations Universal Periodic Review: Ninth Session of the UPR Working Group of the Human Rights Council. Accessed: http://www.reproductiverights.org/document/.

[6] Fowler CI, Gable J, Wang J (2014). Family Planning Annual Report: 2013 national summary.

[7] Fowler CI, Gable J, Wang J, Lasater B (2015). Family Planning Annual Report: 2014 national summary.

[8] Frost JJ, Finer LB, Tapales A (2008). The impact of publicly funded family planning clinic services on unintended pregnancies and government cost savings. J Health Care Poor Underserved.

[9] Gavin L, Moskosky S, Carter M, Curtis K, Glass E, Godfrey E, Marcell A, Mautone-Smith N, Pazol K, Tepper N, Zapata L (2014). Providing Quality Family Planning Services: Recommendations of CDC and the U.S. Office of Population Affairs. MMWR.

[10] Gold RB (2007). Stronger together: Medicaid, title X bring different strengths to family planning effort. Guttmacher Policy Rev.

[11] Institute of Medicine-IOM (2010). Women’s Health Research: Progress, Pitfalls, and Promise.

[12] KFF (2015). Status of state action on the Medicaid expansion decision.

[13] Medicaid (2014). Medicaid chip program information. Available at: https://www.medicaid.gov/medicaid-chip-program-information/program-information/downloads/medicaid-and-chip-eligibility-levels-table.pdf. Accessed 6 Dec 2015.

[14] Monea E, Thomas A (2011). Unintended pregnancy and taxpayer spending. Perspect Sex Reprod Health.

[15] Mosher WD, Jones J, Abma JC (2012). Intended and unintended births in the United States: 1982–2010. National health statistics reports; no 55.

[16] Office of Population Affairs (OPA). (May, 2014) Retrieved March 09, 2016, from http://www.hhs.gov/opa/title-x-family-planning/.

[17] Oglesby WH (2014). Perceptions of and preferences for federally-funded family planning clinics. Reprod Health.

[18] Patient Protection and Affordable Care Act of 2010. Available at: http://www.gpo.gov/fdsys/pkg/PLAW-111publ148/pdf/PLAW-111publ148.pdf. Accessed 1 Mar 2015.

[19] Rosenbaum S, Westmoreland TM (2012). The Supreme Court’s surprising decision on the Medicaid expansion: how will the federal government and states proceed?. Health Aff (Millwood).

